# Supplementary material for: Absence of Seed-Mediated Transmission of Cucumber Mosaic Virus in Espelette Pepper Crops despite Widespread and Recurrent Epidemics
Source: Viruses. 2023 Oct 27;15(11):2159. doi: 10.3390/v15112159 (PMC10674872; doi:10.3390/v15112159)
Supplement: Supplementary file 1 [file viruses-15-02159-s001.zip › viruses-2556724-supplementary.pdf]

Supplementary figures

Absence of seed-mediated transmission of cucumber mosaic virus in Espelette pepper crops despite widespread and recurrent epidemics

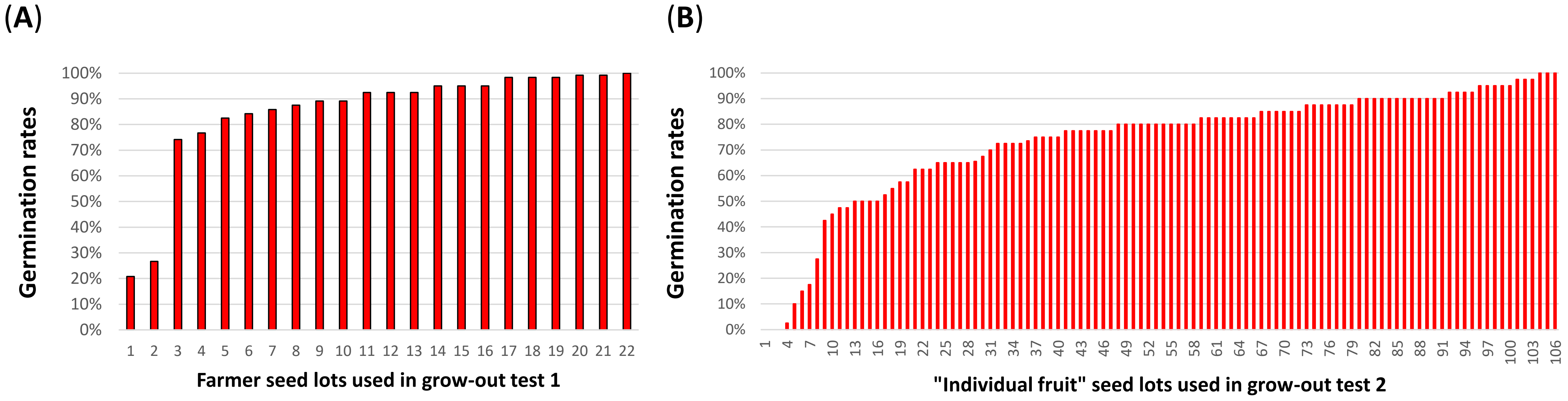

Figure S1. Germination rates of the Gorria pepper seed lots used in this study.

(**A**) Germination rates of the « farmer » seed lots used in grow-out test 1. The mean germination rate for the 22 seed lots derived from farmer mass selection was 85%. The overall germination rate reached 91% when 2 seed lots with low germination rates (of 21 and 27 %) were excluded.

(**B**) Germination rates of the 106 « individual fruit » seed lots used in grow-out test 2.

Each seed lot was the progeny of a single fruit collected during a field survey in the Espelette area in 2021, and diagnosed positive for CMV (based on a DAS-ELISA test). The mean germination rate was 74%.

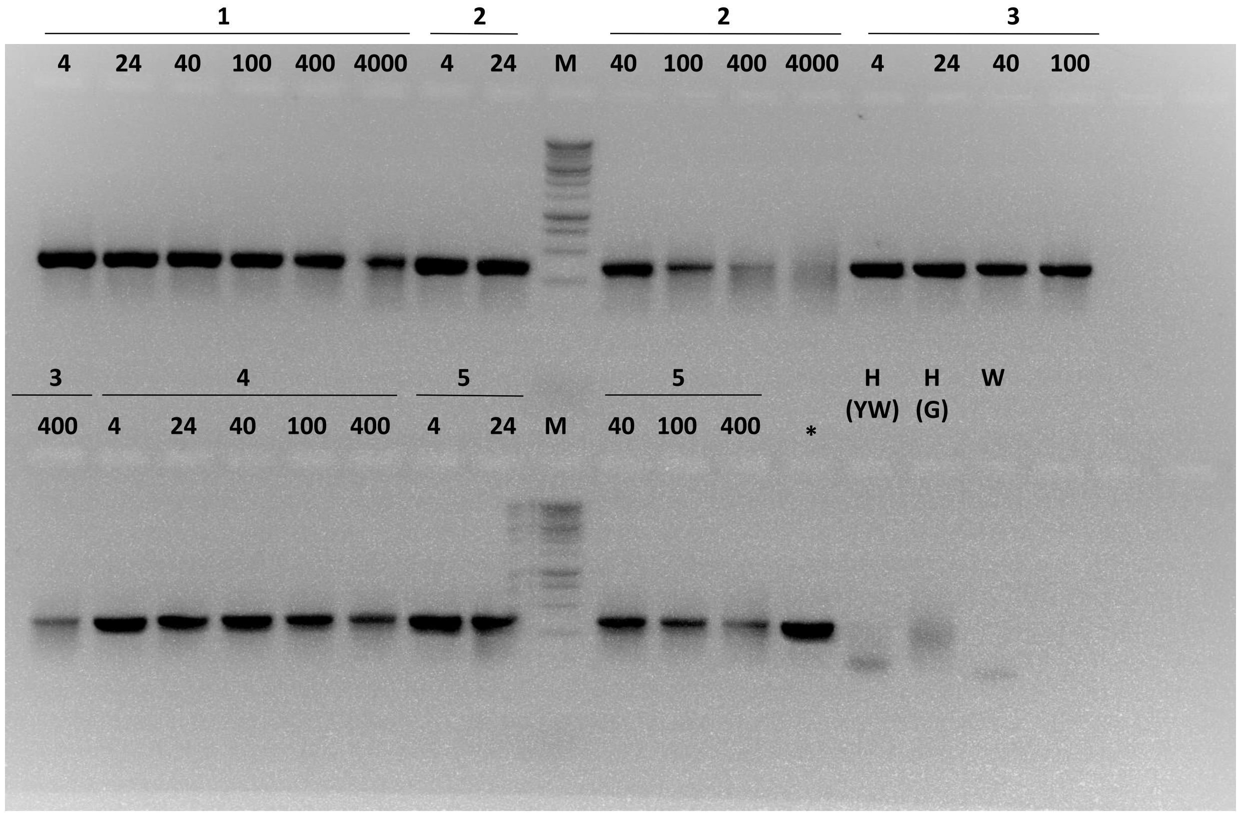

Figure S2. RT-PCR sensitivity assay on pools of infected and healthy Gorria pepper leaves.

RT-PCR analyses were performed on Gorria leaves infected by mechanical inoculation with a CMV isolate corresponding to the main haplotype present on pepper crops in the Espelette area. Infection was ascertained by the appearance of symptoms and a DAS-ELISA test prior to RNA extraction and the RT-PCR test.
Primer pairs described in [10] were used to amplify a 376 nt sequence on CMV genomic RNA 1.
Five microliters of PCR product were loaded on a 1.5% agarose gel.
Sample numbers (1-5) corresponding to 5 different Gorria pepper plants and dilution factors (4-4000, in crude extract from healthy Gorria leaves) are indicated.
Asterisk indicates the positive control (leaves of seedling mechanically infected with CMV).
H (YW), negative control (healthy Yolo Wonder pepper leaves); H (G), negative control (healthy Gorria pepper leaves); W, water (PCR negative control); M, 1 kb DNA ladder (Promega); smallest band: 250, 253 bp; second smallest band: 500 bp.

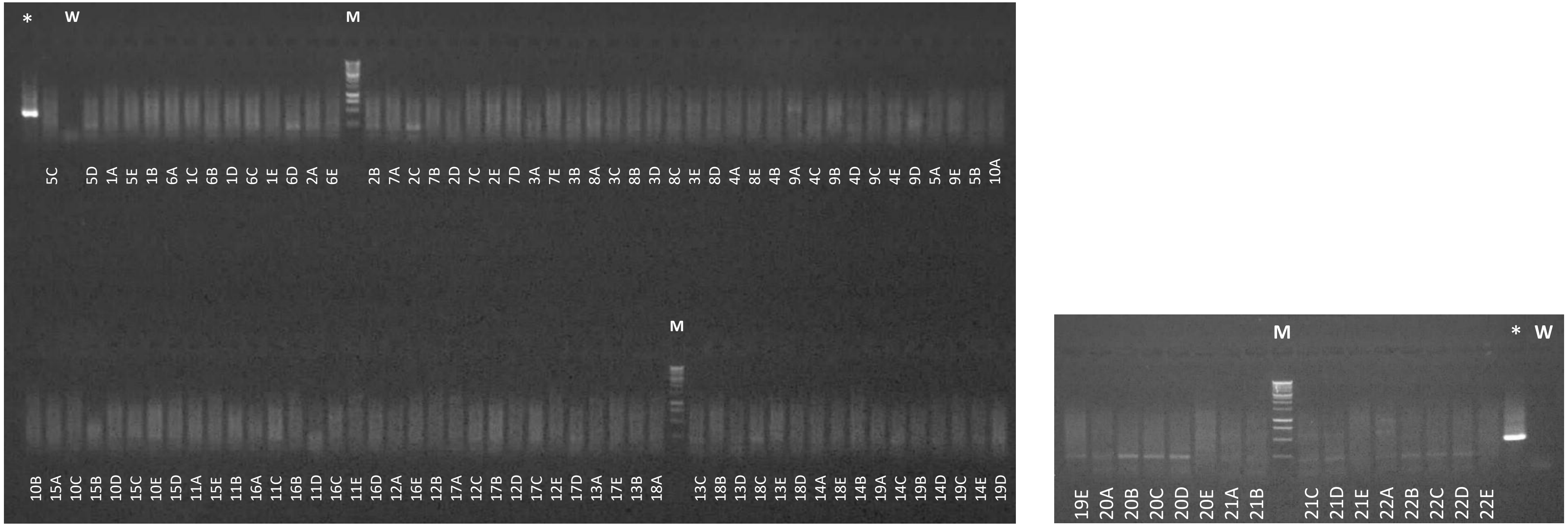

Figure S3. RT-PCR analyses on pooled leaf samples from grow-out test 1.
A total of 22 « farmer » seed lots, numbered 1 through 22, were tested in grow-out test1.
RT-PCR tests were performed on pools of 24 leaves (or less, depending on the number of seedlings that germinated) using CMV genomic RNA 3 primers described in [10].
A total of 5 pooled leaf samples (A-E) was tested per seed lot.
The expected PCR product size is 436 bp.
Positive controls (leaves of seedlings infected with CMV by mechanical inoculation) are indicated by white asterisks.
W, water (PCR negative control); M, 1 kb DNA ladder (Promega); smallest band: 250, 253 bp; second smallest band: 500 bp.

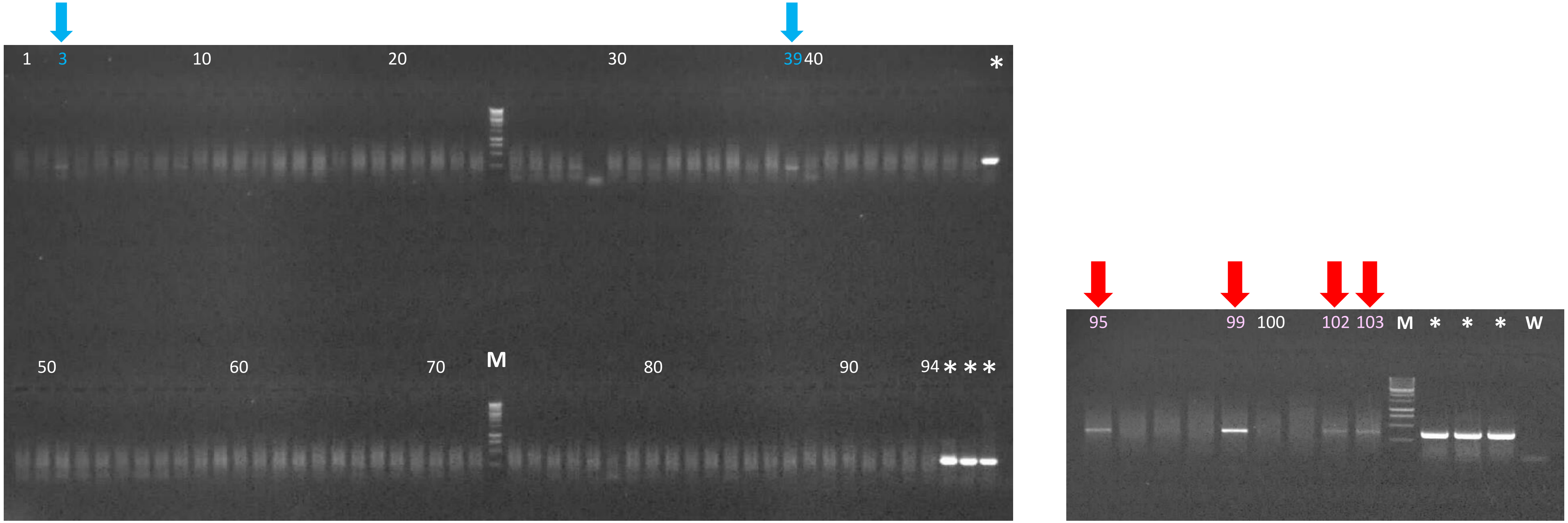

Figure S4. RT-PCR analyses on pooled leaf samples from grow-out test 2.

A total of 106 « individual fruit » seed lots positive for CMV were tested in grow-out test 2.

RT-PCR tests were performed on a single pool of 40 leaves (or less depending on the number of seedlings that germinated) for each seed lot, using CMV genomic RNA 1 primers described in [10]. The expected PCR product size is 376 bp.

Positive controls (leaves of seedlings infected with CMV by mechanical inoculation) are indicated by white asterisks.

W, water (PCR negative control); M, 1 kb DNA ladder (Promega); smallest band: 250, 253 bp; second smallest band: 500 bp.

Because 3 seed lots among the 106 CMV positive seed lots yielded no seedlings, there is a total of 103 RT-PCR results.

Two samples (indicated by blue arrows, samples 3 an 39) displayed very weak bands that were slightly smaller than the expected size. Sequencing of these PCR products showed that the amplicon was not amplified from CMV but corresponded to a plant sequence. These two samples were therefore negative for CMV.

One sample with a strong band and three samples with a weak band of approximately the expected size are indicated by red arrows (samples 95, 99, 102 and 103). These four samples were found to be false positives, resulting from cross-contaminations, as DAS-ELISA and RT-PCR tests performed on new leaf samples collected on the seedlings corresponding to these four bulked leaf samples were all negative (see Figures S5 and S6).

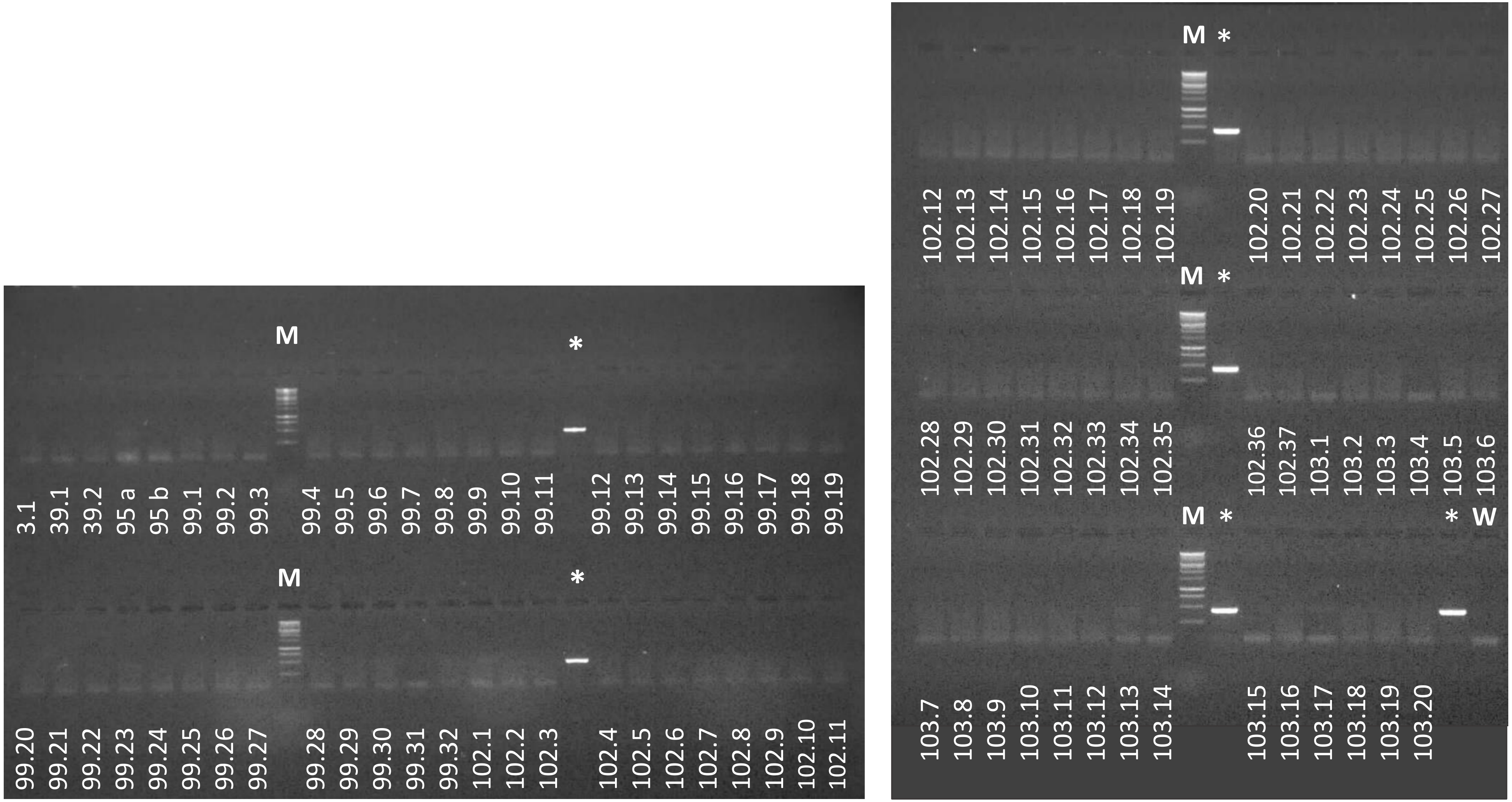

Figure S5. **RT-PCR analyses using CMV genomic RNA 1 primers on individual leaves of seedlings from grow-out test 2.**

RT-PCR tests were performed on individual leaves of a total of 89 seedlings corresponding to 3 seed lots (i.e. the progeny of 3 fruits, samples 99, 102 and 103 on Figure S4) for which initial RT-PCR tests on bulked leaf samples yielded a PCR product of the expected size.

For a fourth seed lot (sample 95 on Figure S4), two bulks (a and b) of respectively 11 and 13 leaves were tested.

Freshly collected leaves corresponding to samples 3 (a single pepper seedling) and 39 (two pepper seedlings) on figure S4 were also included in this test.

CMV was not detected in any of these freshly collected leaf samples.

Positive controls (leaves of seedlings infected with CMV by mechanical inoculation) are indicated by white asterisks.

W, water (PCR negative control); M, 1 kb DNA ladder (Promega); smallest band: 250, 253 bp; second smallest band: 500 bp.

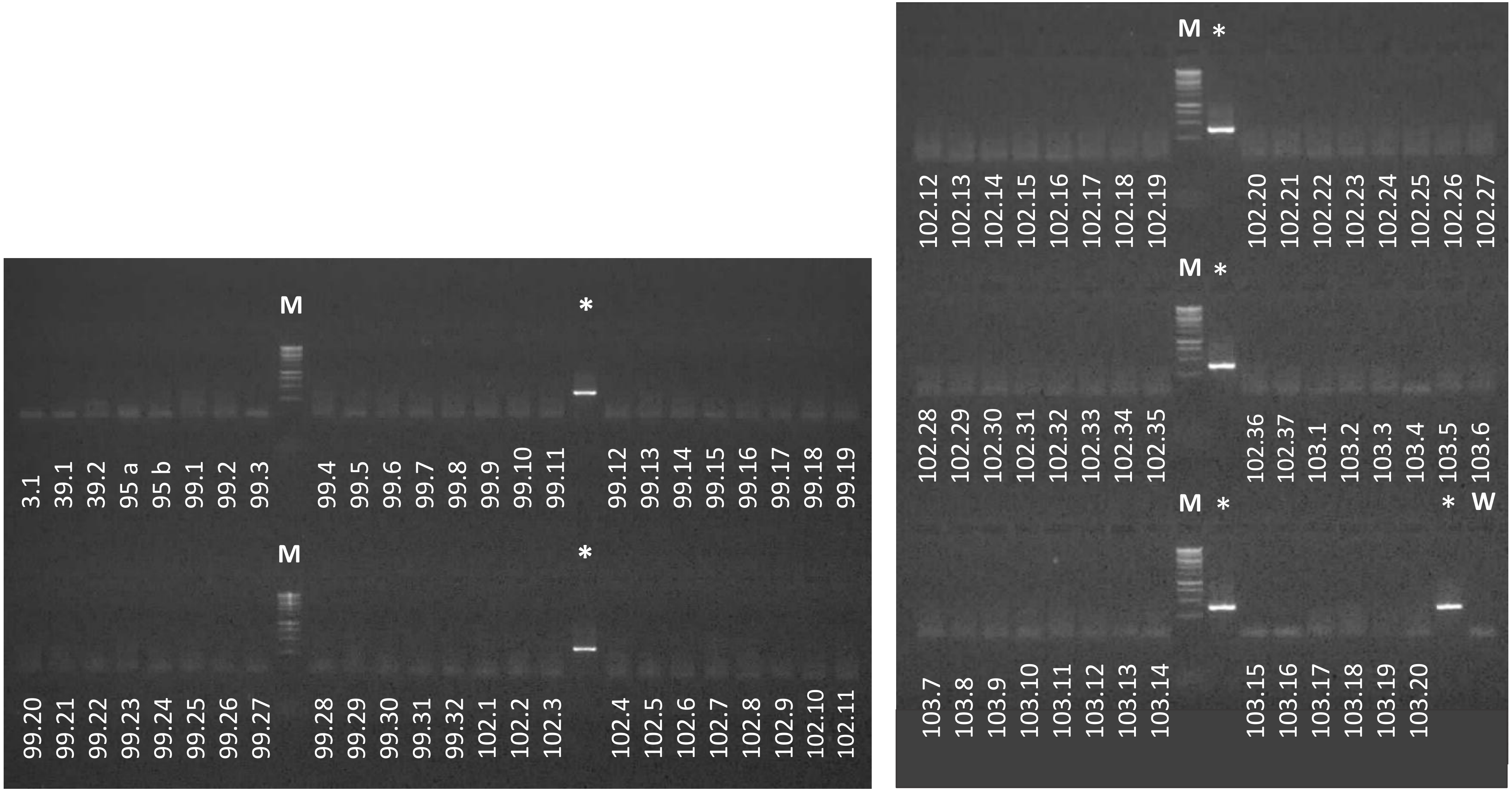

Figure S6. **RT-PCR analyses using CMV genomic RNA 3 primers on individual leaves of seedlings from grow-out test 2.**

RT-PCR tests were performed on individual leaves of a total of 89 seedlings corresponding to 3 seed lots (i.e. the progeny of 3 fruits, samples 99, 102 and 103 on Figure S4) for which initial RT-PCR tests on bulked leaf samples yielded a PCR product of the expected size.

For a fourth seed lot (sample 95 on Figure S4) two bulks (a and b) of respectively 11 and 13 leaves were tested.

Freshly collected leaves corresponding to samples 3 (a single pepper seedling) and 39 (two pepper seedlings) on figure S4 were also included in this test.

CMV was not detected in any of these freshly collected leaf samples.

Positive controls (leaves of seedlings infected with CMV by mechanical inoculation) are indicated by white asterisks.

W, water (PCR negative control); M, 1 kb DNA ladder (Promega); smallest band: 250, 253 bp; second smallest band: 500 bp.
